# Adaption of Seasonal H1N1 Influenza Virus in Mice

**DOI:** 10.1371/journal.pone.0028901

**Published:** 2011-12-16

**Authors:** Lili Xu, Linlin Bao, Fengdi Li, Qi Lv, Yila Ma, Jiangfang Zhou, Yanfeng Xu, Wei Deng, Lingjun Zhan, Hua Zhu, Chunmei Ma, Yuelong Shu, Chuan Qin

**Affiliations:** 1 Institute of Laboratory Animal Sciences, Chinese Academy of Medical Sciences & Comparative Medicine Center, Peking Union Medical Collage; Key Laboratory of Human Disease Comparative Medicine, Ministry of Health; Key Laboratory of Animal Model of Human Diseases, State Administration of Traditional Chinese Medicine, Beijing, China; 2 State Key Laboratory for Molecular Virology and Genetic Engineering, Chinese National Influenza Center, National Institute for Viral Disease Control and Prevention, China Centers for Disease Control, Beijing, China; University of Cambridge, United Kingdom

## Abstract

The experimental infection of a mouse lung with influenza A virus has proven to be an invaluable model for studying the mechanisms of viral adaptation and virulence. The mouse adaption of human influenza A virus can result in mutations in the HA and other proteins, which is associated with increased virulence in mouse lungs. In this study, a mouse-adapted seasonal H1N1 virus was obtained through serial lung-to-lung passages and had significantly increased virulence and pathogenicity in mice. Genetic analysis indicated that the increased virulence of the mouse-adapted virus was attributed to incremental acquisition of three mutations in the HA protein (T89I, N125T, and D221G). However, the mouse adaption of influenza A virus did not change the specificity and affinity of receptor binding and the pH-dependent membrane fusion of HA, as well as the *in vitro* replication in MDCK cells. Notably, infection with the mouse adapted virus induced severe lymphopenia and modulated cytokine and chemokine responses in mice. Apparently, mouse adaption of human influenza A virus may change the ability to replicate in mouse lungs, which induces strong immune responses and inflammation in mice. Therefore, our findings may provide new insights into understanding the mechanisms underlying the mouse adaption and pathogenicity of highly virulent influenza viruses.

## Introduction

Seasonal influenza A viruses can cause acute respiratory infections with high morbidity and considerable mortality, particularly in children and the elderly [1]. The disease is characterized by a sudden onset of malaise and fever, followed by upper and sometimes lower respiratory signs, myalgia, and headache [2]. Systemic disease manifestations subside once the virus is cleared, usually within three to five days after the infection, but respiratory signs including coryza and cough may persist longer [2]. Severe diseases and mortality occur preferentially in immunocompromised patients and individuals with pre-existing lung diseases, and are often due to secondary bacterial infections [3]. However, the pathogenic process of influenza virus infection and related immune responses are not fully understood.

The mouse model of influenza is an excellent model for studying the pathogenesis of influenza virus because mice infected with influenza can develop pneumonia, pathologically similar to that in humans [4]. Experimental infection of mouse lungs with influenza virus has provided insights into understanding viral pathogenicity and adaption [5]. Notably, mice are naturally insusceptible and insensitive to infection with influenza viruses and mice infected with newly isolated human influenza A viruses usually become asymptomatic. Many strains of mice can be infected experimentally with influenza viruses, particularly with mouse lung-adapted viruses [6], and allow the infected viruses to replicate in their lungs [5]. Following infection with influenza A virus, the virus induced humoral immunity can clear the viruses in the lungs around five days post infection. However, mice infected with the mouse-adapted influenza viruses can display pathogenic inflammation in the bronchi and lungs, leading to alveolitis and lethal pneumonitis, similar to that in humans [4], [7]. Hence, the changes in the viruses during mouse adaptation may provide new insights into understanding factors contributing to the development of virus-related lung inflammation in humans. Furthermore, adaption of human influenza virus to mice by serial passages can result in genetic variants with the mutations in multiple genes, such as hemagglutinin (HA), which is a primary factor of mouse lung virulence because of its receptor binding and host membrane fusion activities [8], [9], [10], [11], [12], [13], and other genes for M, PA, PB1, PB1-F2, PB2, and NS1 [12], [13], [14], [15], [16], [17], [18], [19], [20], [21], [22], [23], [24]. Previous studies have shown that mouse-adapted A/FM/1/47(H1N1) (FM-MA) from 12 sequential mouse-lung passages has a high ability to replicate and virulence [9], which is associated with the mutations of Gly-to-Try at residue 47 of the HA2 subunit and Thr-to-Ala at residue 139 of the matrix protein [13]. Further studies indicate that the increased virulence to mice is controlled by both mutations, whereas the enhanced replication in Madin-Darby canine kidney (MDCK) cells is attributed to the mutation in the matrix protein [13].

In the present study, the prototype seasonal H1N1, A/Brisbane/59/2007, without a prior history of mouse passage, was used to generate virulent variants by serial mouse-lung passages to identify the potential mutations associated with virulence and viral infection-related inflammatory responses in mice. We found that the mouse adaption not only directly affected viral properties, but also indirectly modulated the host defense system. Therefore, our findings may provide new insights into the pathogenesis of infection with highly virulent strains of influenza and related inflammation. We discussed the implications of our findings.

## Materials and Methods

### Viruses and cells

The seasonal H1N1 influenza virus A/Brisbane/59/2007 (the third passage in the allantoic cavities of 10-day-old chicken eggs) was kindly provided by Dr. Honglin Chen (Hongkong University). The virus was subsequently inoculated in the allantoic cavities of 10-day-old chicken eggs and cultured at 37°C for 48 h, and aliquots were stored at −80°C. MDCK cells were maintained in Dulbecco's modified Eagle's medium (DMEM, Invitrogen, Carlsbad, USA) supplemented with 10% FBS. All experiments involving the mouse-adapted variants of the seasonal H1N1 influenza virus were conducted under biosafety level 3 (BSL-3) conditions.

### Adaption of seasonal H1N1 influenza viruses in mice

Mouse-adapted variants of A/Brisbane/59/2007 virus were derived by eight sequential mouse lung-to-lung passages. Briefly, female 5-week-old BALB/c mice were obtained from the Institute of Laboratory Animal Sciences, Beijing, and housed in a specific pathogen free facility. Individual mice were anesthetized with tribromoethanol and inoculated intranasally with 50 µl of allantoic fluid containing wild-type A/Brisbane/59/2007 virus. Three days later, their lungs were collected, homogenized, and centrifuged. Subsequently, 50 µl of the supernatants were used for the next passage. After a total of 8 passages, the viruses in the lung homogenates were further cloned once by plaque purification in MDCK cells, and the cloned virus was passaged once in the allantoic cavities of 10-day-old chicken eggs at 37°C for 48 h to prepare a virus stock. The experimental protocol was evaluated and approved by the Institute of Animal Use and Care Committee of the Institute of Laboratory Animal Science, Peking Union Medical College (ILAS-PC-2010-002).

### Determination of viral infectivity

The infectivity of wild-type and mouse-adapted seasonal H1N1 viruses was determined in MDCK cells by plaque assay and expressed as log_10_ plaque-forming units (PFU) per milliliter. Briefly, confluent MDCK cells were inoculated with 10-fold serial dilutions of virus at 37°C for 1 h. The cells were then washed and cultured with minimal essential medium (MEM) containing tosylsulfonyl phenylalanyl chloromethyl ketone (TPCK)-treated trypsin (0.5 µg/ml) and antibiotics (Sigma, St Louis, USA) at 37°C for 48 h, and the formed plaques were visualized by neutral red staining.

### DNA sequencing and alignment

All gene segments of each passage of the viruses in the supernatants of homogenized lungs of mice were amplified by high fidelity PCR (KOD plus DNA polymerase, Toyobo, Japan). The PCR products were purified and cloned into a pGEM-T easy vector (Promega, Madison, USA). A total of five positive clones for each gene segment of individual types of viruses were selected randomly for sequencing (Invitrogen, Shanghai, China). The resulting sequences of all viruses were analyzed and aligned using ClustalW software (version 1.83).

### Replication kinetics in MDCK cells

To determine multistep growth curves of the virus *in vitro*, MDCK cells in six-well plates were infected in duplicate with 25 PFU of wild-type and mouse-adapted viruses, respectively. After 60 min absorption at 37°C, the cells were washed and cultured with 3 ml of serum-free MEM containing TPCK-treated trypsin (0.5 µg/ml) and antibiotics for 72 h. The cell supernatants (100 µl) were collected at 0, 12, 24, 36, 48, 56, and 72 h post infection and centrifuged at 3000× *g* for 10 min, followed by titrating by PFU assay and storing at −80°C.

### Pathogenicity of virus in BALB/c mice

To compare the pathogenicity of wild-type with mouse-adapted viruses *in vivo*, female 5-week-old BALB/c mice (n = 20 per group) were anesthetized and inoculated intranasally with 50 µl (10^4.2^ PFU) of each type of virus, respectively. Ten mice were selected randomly from each group for monitoring the signs of disease, weight loss, and mortality daily up to 14 days post inoculation (d.p.i.). The remaining ten mice in each group were euthanized at 4 d.p.i. and their blood samples, bronchoalveolar lavage fluid (BALF), and lung tissues were collected for subsequent analysis of viral replication, lung histology, pro-inflammatory cytokines, hemostatic parameters, and T lymphocytes counts.

### Real-time PCR

Total RNA was isolated from individual BALF samples using the RNeasy Mini Kit, according to the manufacturer's instructions (Qiagen, Hilden, Germany). The RNA was reversely transcribed into cDNA using random primers and a SuperScript II reverse transcriptase (200 U) reaction mixture (20 µl) (Invitrogen). The target gene mRNA transcripts were determined by RT-PCR using a SYBR Green PCR Master Mix, the specific primers, and a StepOne PCR system (ABI, USA). The sequences of primers were forward 5′-ctgagaagcagatactgggc-3′ and reverse 5′-ctgcattgtctccgaagaaat-3′ for HA gene fragment (340 bp), using GAPDH gene fragment amplification as the internal reference control. The PCR amplifications were performed in duplicate at 94°C for 3 min, and subjected to 35 cycles of 94°C for 30 s, 55°C for 30 s, and 72°C for 30 s.

### Pathological analysis

Immediately following euthanasia, the mouse lungs were removed, inflated, and fixed with 10% neutral buffered formalin overnight at 4°C. Subsequently, the formalin-preserved lung samples were embedded in paraffin and sectioned. Serial lung tissue sections at 4-µm were stained with Hematoxylin and Eosin (H&E), and examined for pathological changes under a light microscope (Olympus BX-50).

### Receptor-binding assay

Synthetic 3′-sialyl-*N*-acetyllactosamine (3′SLN-PAA)-Biotin, 3′SLN-DI-PAA-Biotin (double saccharide connection in series), 6′-sialyl-*N*-acetyllactosamine (6′SLN-PAA)-Biotin, and 6′SLN-DI-PAA-Biotin (double saccharide connection in series) were provided by the Scripps Research Institute (La Jolla, CA, USA). The receptor binding procedure was carried out as described elsewhere, with some modifications [25]. Briefly, sialosaccharides at 0–10 µg/ml were coated on 96-well flat-bottom polystyrene plates and allowed to attach overnight at 4°C. The plates containing sialylosaccharides were crosslinked under a UV light at 254 nm for 10 min and after washed five times with PBS, the plates were blocked with 2% skim dry milk in PBS for 8 h at 4°C. After washed five times with 0.1% Tween-20 PBS, individual wells in the plates were added in sextuplicate with virus culture supernatants (50 µl) containing 32 HAU and incubated on ice overnight, respectively. After washing, the bound viruses were detected with a mouse monoclonal antibody against the A/Brisbane/59/2007 strain (Sino Biological, Beijing, China) and visualized by horseradish peroxidase (HRP)-conjugated anti-mouse IgG antibodies and tetramethylbenzidine substrate solution, followed by measuring absorbance at 450 nm. A/Shenzhen/406H/2006 (H5N1) and A/Brisbane/10/2007 (H3N2) viruses were used as positive controls for 3′SLN and 6′SLN bindings, respectively.

### Hemolysis assay

Hemolysis assay was performed in 96 well round bottom microtiter plates. Briefly, 25 µl of each type of virus (640 HAU) was reacted in triplicate with 50 µl of the 2% pre-washed turkey erythrocytes at 4°C for 10 min and centrifuged (1000× *g* for 2 min). After removing the supernatants, the virus and erythrocyte mixture was cultured with 75 µl of 100 mM citrate buffers with varying pH values from 4.8 to 6.0 in increments of 0.1 pH unit at 37°C for 1 h and centrifuged. Subsequently, their supernatants were transferred to flat bottom ELISA plates for measuring at 540 nm [26]. The FM-MA (H1N1) virus was used as a positive control to validate the hemolysis assay, as compared with the data published previously (15, 31). Meanwhile, *Escherichia coli* was used as a virus-free negative control.

### Hematology analysis

The total white blood cell (WBC) counts and lymphocytes in individual heparinized blood samples were determined on an ACT TM laser-based hematology analyzer (Beckman Coulter, USA).

### Flow cytometry analysis

Peripheral blood samples were collected from individual mice and peripheral blood mononuclear cells were prepared. The cells were treated with the purified anti-CD16/32 and stained with FITC-anti-CD3, PE-anti-CD4, or APC-anti-CD8, respectively. The frequency of different subsets of T cells was determined by flow cytometry (FACS CANTO, BD, USA).

### Quantification of cytokines and chemokines

The concentrations of granulocyte colony-stimulating factor (G-CSF), granulocyte-macrophage colony-stimulating factor (GM-CSF), interferon-γ (IFN-γ), monokine induced by IFN-γ (MIG), interleukin-1α (IL-1α), IL-1β, IL-4, IL-6, IL-10, IL-12/IL-23p40, IL-13, IL-17, IL-21, monocyte chemoattractant protein 1 (MCP-1), macrophage inflammatory protein 1α (MIP-1α), MIP-1β, tumor necrosis factor (TNF), and keratinocyte-derived chemokine (KC) in serum and BALF samples were determined by flow cytometry (FACS CANTO, BD, USA) using the Cytometric Beads Array (CBA) Kits, according to the manufacturers' instructions (BD, USA). Briefly, 50 µl of each testing sample were labeled in duplicate with equal volumes of diluted FlexSet capture beads at room temperature for 1 h and treated with PE-conjugated detection reagent. After washing, the captured cytokines and chemokines were analyzed by flow cytometry.

### Statistical analysis

Data are expressed as mean ± SD. The difference among different groups was analyzed by one-way ANOVA and post hoc. Bonferroni correction analysis and the difference between two groups were analyzed by Student's *t*-test using SPSS 11.5 software. A probability value of <0.05 was considered as statistically significant.

## Results

### Adaption of seasonal H1N1 virus in mice

**Figure 1:**
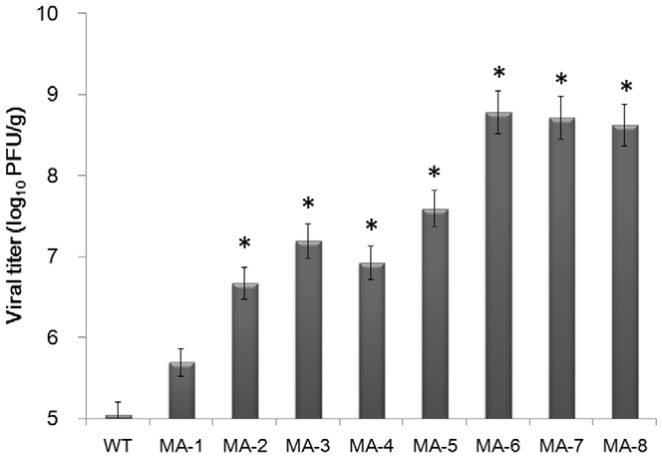
Adaption process of each passage of mouse-adapted seasonal H1N1 A/Brisbane/59/2007 viruses in mouse lungs. BALB/c mice were infected with wild-type virus and three days later, their lungs were dissected out to generate the first passage of mouse-adapted virus (MA-1). Subsequently, the lung lysates were used for infection of mice to generate MA-2, which was subjected serial mouse lung passages (total of eight passages). The replication of each passaged virus was determined as viral titers in the lungs. Data are expressed as mean ± SD of viral titers (log10 PFU/mg) in the lungs of each group of mice (n = 3 per group) from three separate experiments. * *P*<0.05 vs. the values of wild type virus.

Seasonal H1N1 influenza viruses are almost avirulent to mice. To increase the virulence of H1N1 influenza virus, individual mice were inoculated intranasally with 10^6.2^ PFU A/Brisbane/59/2007 virus, and three days later, their lungs were dissected out and the supernatants of lung homogenates were used for continual eight passages to generate mouse-adapted variants. While inoculation with wild-type (WT) and mouse-adapted A/Brisbane/59/2007 from passage 1 to 2 (MA-1 to MA-2) did not result in obvious clinical symptoms and lung lesions in mice, the viral titers in the lung tissues increased from 5.05 to 6.67 log_10_ PFU/g. The mice inoculated with MA-3 to MA-5 developed clinical symptoms, including hunched posture and ruffled fur, and displayed large lesions in the lung tissues, accompanied by increasing viral titers to 7.19–7.59 log_10_ PFU/g. Some mice inoculated with MA-6 were dead while all of the mice inoculated with MA-8 died or were in agonal states, accompanied by defuse inflammation in the lungs and increasing viral titers to 8.78-8.62 log_10_ PFU/g, indicating high virulence and pathogenicity (Table 1 and Fig. 1).

**Table 1:** Clinical characteristics of mice.

Virus	Death	Impending death	Clinical symptoms of alive	Pathological change of lung tissues
WT	0	0	None	None
MA-1	0	0	None	None
MA-2	0	0	2/3 hunched posture and ruffled fur	None
MA-3	0	0	3/3 hunched posture and ruffled fur	1/3 appeared nidus near hilum of lung
MA-4	0	0	3/3 hunched posture, ruffled fur	3/3 large area of lungs appeared lesions
MA-5	0	0	3/3 hunched posture, ruffled fur	3/3 large area of lungs appeared lesions
MA-6	1	0	2/2 hunched posture, ruffled fur	2/2 large area of lungs appeared lesions
MA-7	1	1	2/2 hunched posture, ruffled fur	2/2 all area of lungs appeared lesions
MA-8	2	1	1/1 hunched posture, ruffled fur	1/1 all area of lung appeared lesions

Note: WT, wild-type; MA, mouse-adapted and the number represents the passage. Groups of mice (n = 3 per group) were inoculated with each type of wild-type or mouse-adapted virus and monitored for three days. Subsequently, the mouse lungs were collected and the supernatants of lung homogenates were used for serial passages.

### Sequence analysis of mouse-adapted H1N1 viral genomes

The full-length sequences of each passage of viruses in the supernatants of homogenized lungs of mice were obtained by high fidelity PCR and cloned. Subsequently, five positive clones were selected randomly from individual libraries for sequencing. Genomic mutations in those mouse-adapted viruses are listed in Table 2. Identical sequences were detected from five clones of each gene segment and the mutations of all virus isolates were located in the HA coding sequence. While the MA-2, MA-3, and MA-6 gradually obtained the HA mutations at position of 125 (Asn-to-Thr), 221 (Asp-to-Gly), and 89 (Thr-to-Ile), respectively, there were no genomic substitutions between MA-6 and MA-8 viruses. Furthermore, there was no synonymous mutation detected in any of the genome segments during the process of mouse lung adaption. The sequences of the HA gene in wild-type and MA-2, MA-3, MA-8 viruses were deposited in GenBank under accession numbers of JN899402, JN899403, JN899404, and JN899405.

**Table 2:** Sequence alignment of wild-type and mouse-adapted seasonal H1N1 A/Brisbane/59/2007 viruses of each passage.

Virus	PB2	PB1	PA	HA						NP	NA	M2&M1	NEP&NS1
				nt	aa	nt	aa	nt	aa				
				317	89	425	125	713	221				
WT	-	-	-	C	Thr	A	Asn	A	Asp	-	-	-	-
MA-1	-	-	-	C	Thr	A	Asn	A	Asp	-	-	-	-
MA-2	-	-	-	C	Thr	**C**	**Thr**	A	Asp	-	-	-	-
MA-3	-	-	-	C	Thr	**C**	**Thr**	**G**	**Gly**	-	-	-	-
MA-4	-	-	-	C	Thr	**C**	**Thr**	**G**	**Gly**	-	-	-	-
MA-5	-	-	-	C	Thr	**C**	**Thr**	**G**	**Gly**	-	-	-	-
MA-6	-	-	-	**T**	**Ile**	**C**	**Thr**	**G**	**Gly**	-	-	-	-
MA-7	-	-	-	**T**	**Ile**	**C**	**Thr**	**G**	**Gly**	-	-	-	-
MA-8	-	-	-	**T**	**Ile**	**C**	**Thr**	**G**	**Gly**	-	-	-	-

Note: WT, wild-type; MA, mouse-adapted; nt, nucleotide; aa: amino acid.

### Growth characteristics of mouse-adapted H1N1 viruses in MDCK cells

**Figure 2:**
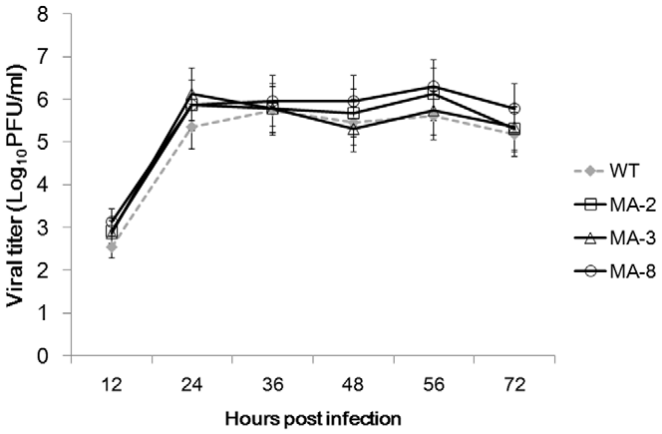
The replication kinetics of wild-type and mouse-adapted seasonal H1N1 A/Brisbane/59/2007 viruses in MDCK cells. MDCK cells were infected in triplicate with 25 PFU of each virus, respectively. The viral yields (log10 PFU/ml) were measured at 12, 24, 36, 48, 56, and 72 h.p.i. Data are expressed as mean ± SD of viral titers (log10 PFU/mg) of each group of cells from three separate experiments.

To evaluate the replication ability of the mouse-adapted seasonal H1N1 A/Brisbane/59/2007 viruses, we assayed the viral yields of three representative strains (MA-2, MA-3, and MA-8) with a respective wild-type strain after multiple replication cycles in MDCK cells. Analysis of replication kinetics showed that the titer of wild-type virus was 10^2.54^ PFU at 12 h.p.i, and then rose to 10^5.358^ at 24 h.p.i and maintained in 10^5^ until 72 h.p.i. The three mouse adapted viruses showed the growth kinetics similar to that of the wild-type virus, and there was no significant difference in the viral titers and growth rates between wild-type and mouse-adapted viruses (*P*>0.05, Fig. 2).

### Pathogenicity of mouse-adapted H1N1 viruses *in vivo*

**Figure 3:**
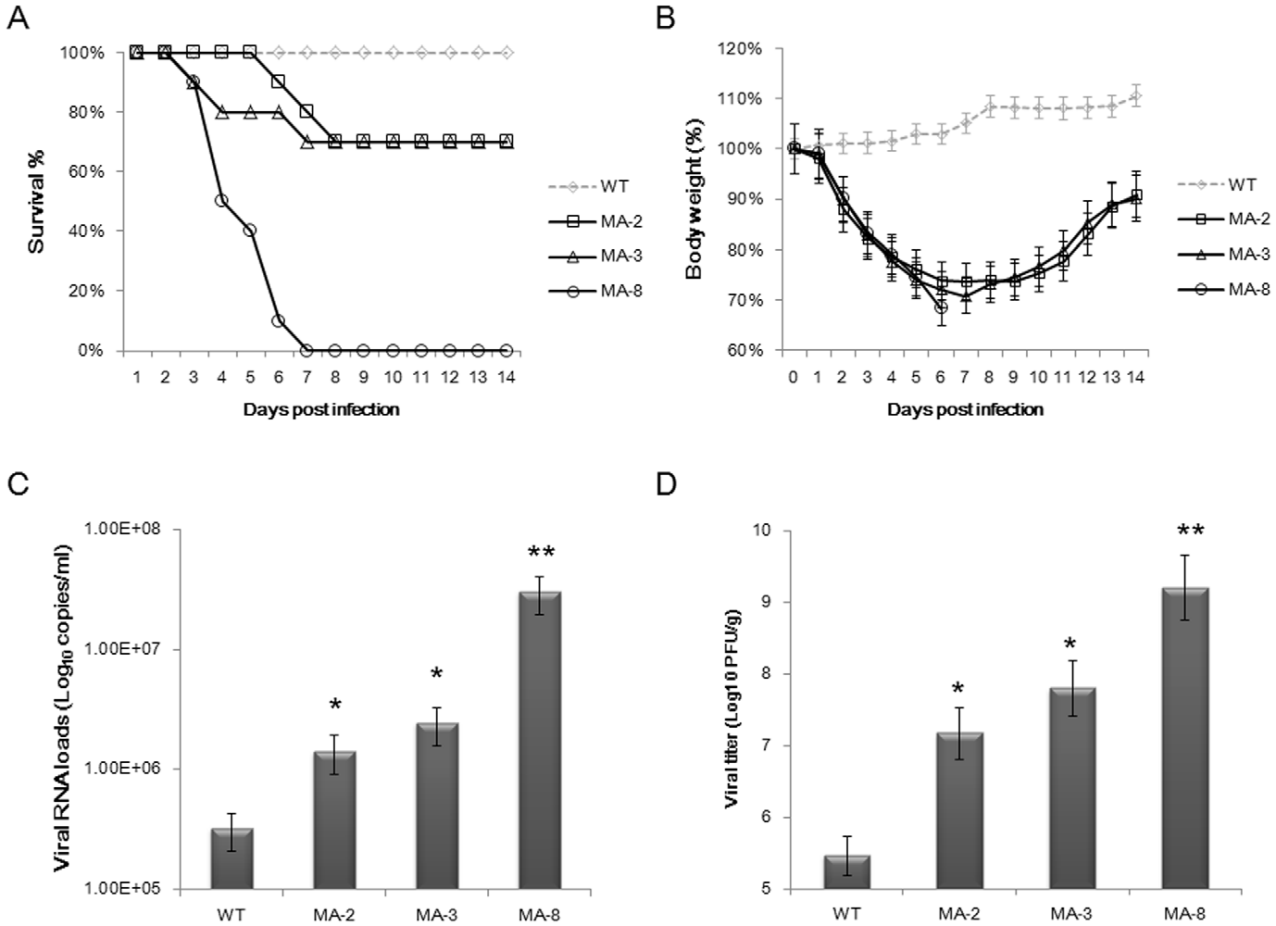
The pathogenicity of wild-type and mouse-adapted seasonal H1N1 A/Brisbane/59/2007 viruses in BALB/c mice. Groups of mice were inoculated intranasally with each type of virus (n = 20 per group; 50 µl of 10^4.2^ PFU) and ten mice were randomly selected and monitored daily for signs of disease and mortality up to 14 d.p.i. The remaining ten mice from each group were sacrificed on 4 d.p.i., and their lung tissue and bronchoalveolar lavage fluid (BALF) samples were used for determining the viral RNA and titers as well as histological examination. (A) Survival percentage of mice. (B) Body weight changes of infected mice. Data are expressed as mean % ± SD of each group of mice relative to the value (100%) before inoculation. (C) Viral RNA loads in BALF. Data are presented as mean ± SD of viral loads per milliliter of each group of mice. (D) Viral titers in lung tissues. Data are presented as mean ± SD of log_10_ PFU/mg from each group of mice. * *P*<0.05 or ** *P*<0.01 vs. the values of wild type virus.

To compare the virulence and pathogenicity of the wild-type with mouse-adapted seasonal H1N1 A/Brisbane/59/2007 viruses *in vivo*, BALB/c mice were infected intranasally with WT, MA-2, MA-3, and MA-8 viruses, respectively, and their mortality, mean survival days, total weight loss, viral RNA loads in BALF, and viral titers in lung tissues were evaluated. The percentages of mice that survived during the observation period are shown in Figure 3A. All of the mice infected with WT virus survived. In contrast, only 70% of the mice inoculated with MA-2 and MA-3 viruses survived on 14 d.p.i, and the mean survival days [27] were 11.6 and 10.9, respectively. However, all of the mice infected with MA-8 died on 7 d.p.i with a mean survival period of 3.9 days, accompanied by 32% body weight loss. The loss of body weight in the mice inoculated with MA-2 and MA-3 viruses were less than 30% on 7 d.p.i, after which the mice began to steadily regain their body weight over the remaining observation period. In contrast, the body weight in the mice infected with WT virus continued to increase throughout the observation period (Fig. 3B).

To further investigate the pathogenicity of the wild-type and mouse-adapted seasonal H1N1 A/Brisbane/59/2007 viruses, we determined the virus loads in BALF and lung tissues of mice at 4 d.p.i (Fig. 3C and D). Mice inoculated with MA-8 virus presented with the highest numbers (3.02×10^7^ copies/ml) of virus in BALF, which was significantly higher than that in the mice infected with the other three viruses (*P*<0.05). However, the RNA copy numbers in BALF of the mice infected with other two mouse-adapted viruses (MA-2, MA-3) were significantly higher than that in the mice infected with the wild-type virus (3.20×10^5^ copies/ml, *P*<0.05). Similarly, mice infected with MA-8 exhibited the highest titer (9.21 PFU/g) of virus in their lung tissues, which was significantly higher than that with WT (5.46 PFU/g), MA-2 (7.17 PFU/g), and MA-3 (7.80 PFU/g) (*P*<0.05).

**Figure 4:**
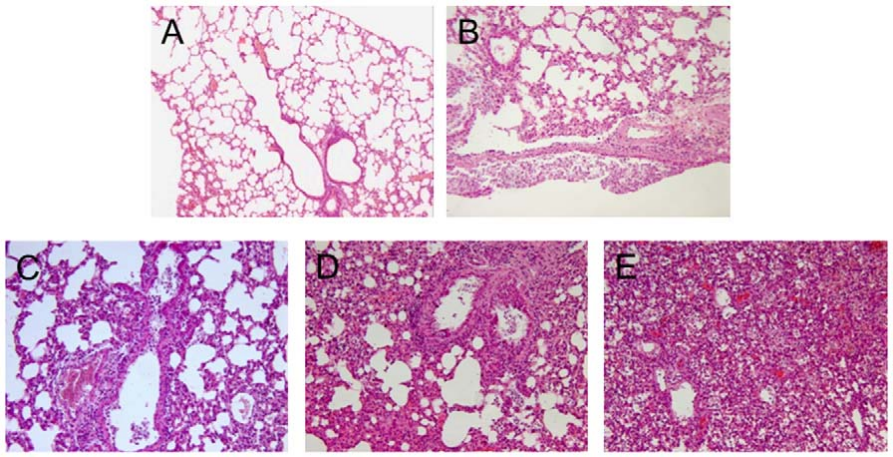
Histological analysis of the lung tissues. The collected lung tissue samples from each group of mice at 4 d.p.i. were subjected to routine histological process and the tissue sections were stained with H&E. Data show are representative images (magnification ×100) of each group of mice (n = 10 per group). (A) The lung tissue section from PBS mock infected mice; (B) The lung tissue section from the mice infected with WT virus; (C) The lung tissue section from the mice infected with MA-2 virus; (D) The lung tissue section from the mice infected with MA-3 virus; (E) The lung tissue section from the mice infected with MA-8 virus.

Characterization of inflammation in the lungs revealed that all lung tissue samples exhibited characteristic pathology of influenza infection, including inflammatory hyperaemia, hemorrhage, edema, and exudative pathological changes. Mice harboring MA-8 virus exhibited the most robust pathological lesions occurring in 100% of the lung tissues (Fig. 4).

### Effect of the mutations in the HA on receptor binding ability and pH optimum fusion

**Figure 5:**
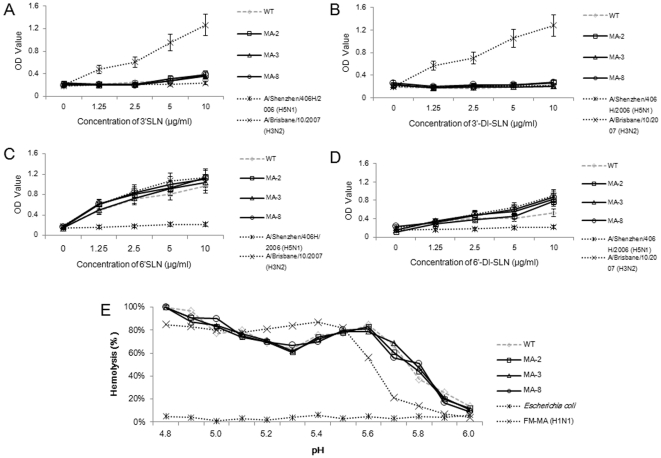
The mouse-adapted virus has receptor binding and pH-dependent fusion properties similar to the wild-type virus. The specificity and affinity of receptor binding and the pH-dependent hemolysis of the mouse adapted and wild-type viruses were determined by in vitro binding to the synthetic biotinylated sialylglycopolymers and pH dependent hemolysis, respectively. Data are expressed as mean ± SD of OD values or percentage of lysing cells by each type of virus. The receptor binding profiles of A/Shenzhen/406H/2006 (H5N1) and A/Brisbane/10/2007 (H3N2) viruses, and the hemolysis profile of FM-MA (H1N1) virus and *Escherichia coli* are shown as controls. (A) Binding to the 3′SLN. (B) Binding to the 3′DI-SLN. (C) Binding to the 6′SLN. (D) Binding to the 6′DI-SLN. (E) The percentage of lysing cells.

Previous studies have suggested that HA mutations may modulate the affinity of HA binding to host sialyl receptors to increase virus virulence [28], [29], [30]. Accordingly, we examined the binding abilities of WT, MA-2, MA-3, and MA-8 to synthetic sialic substrates *in vitro*. As expected, there was no wild-type and mouse-adapted seasonal H1N1 A/Brisbane/59/2007 viruses binding to the avian-type of 3′SLN or 3′DI-SLN receptors (Fig. 5A and B). Furthermore, there was no significant difference in the bindings of virus to the human-type of 6′SLN and 6′DI-SLN receptors among those four types of H1N1 viruses (Fig. 5C and D). Two control viruses, A/Shenzhen/406H/2006 (H5N1) and A/Brisbane/10/2007 (H3N2), selectively bound to 3′ and 6′ substrates, verifying the specificity and validity of the sialyl receptor binding assay.

Next, we determined the optimum pH of fusion for WT, MA-2, MA-3, and MA-8 using hemolysis as an indicator of fusion. The results showed that there were no significant difference in the fusion properties from pH 4.8 to 6.0 between WT and MA viruses (Fig. 5E). These data indicated that the mutations in the HA in mouse-adapted seasonal H1N1 A/Brisbane/59/2007 viruses did not significantly change the specificity and affinity of their binding to the receptors and the abilities of the virus to promote host membrane fusion *in vitro*. The hemolysis profile of FM-MA (H1N1) virus was in accordance with the data published previously [15], [31], but *Escherichia coli* failed to fuse with the turkey erythrocytes, which verified the validity of the hemolysis assay.

### Analysis of peripheral white blood cells in the challenged mice

**Figure 6:**
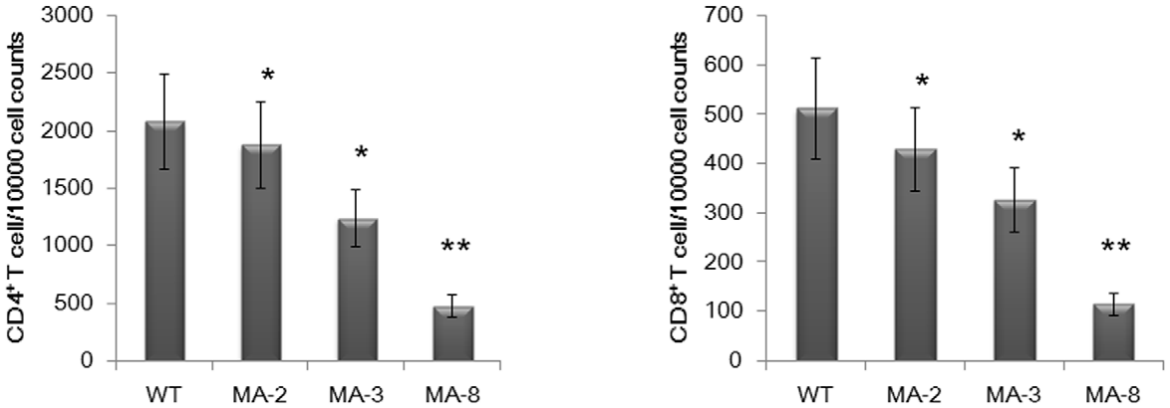
The frequency of peripheral blood CD4^+^ and CD8^+^ T lymphocytes. Peripheral blood samples were obtained from individual mice at 4 d.p.i and their PBMC were prepared. Subsequently, those PBMC were stained with fluorescent anti-CD4 or anti-CD8 and the frequency of CD4^+^ and CD8^+^ T cells were determined by flow cytometry analysis. Data are expressed as mean ± SD of percentages of CD4^+^ or CD8^+^ T cells in each group of mice (n = 10 per group) from three separate experiments. * *P*<0.05 and ** *P*<0.01 vs. the values in the mice infected with wild type virus.

It has been reported that infection with influenza viruses can cause leukopenia [32], [33]. To determine the extent to which infection with wild-type and mouse-adapted viruses induced leukopenia in mice, peripheral blood leukocytes in different groups of mice were counted (Table 3). There was no significant difference in the percentage of neutrophils among the different groups of mice. However, in comparison with those in the mice infected with wild-type virus, the total numbers of white blood cells (WBCs) in the mouse-adapted viruses infected mice were reduced significantly (*P*<0.05), particularly in the mice infected with MA-8 (*P*<0.01) at 4 d.p.i. Significantly reduced numbers of lymphocytes and monocytes were also observed in the mice infected with mouse-adapted viruses. Analysis of the frequency of CD4^+^ and CD8^+^ T cells revealed significantly lower frequency of CD4^+^ and CD8^+^ T cells in the mice infected with mouse adapted viruses at d.p.i (Fig. 6). Therefore, infection with the mouse-adapted viruses, particularly with MA-8, induced severe leukopenia in mice.

**Table 3:** Impact of viral infection on the mouse lymphocyte populations in whole blood.

Virus	WBC^a^	% LY^b^	% NE^b^	% MO^b^
None	10.3	76.7	36.6	8.3
WT	9.4	64.9	34.2	9.5
MA-2	5.9*	58.3*	36.2	7.4*
MA-3	5.4*	54.4*	47.4	8.9*
MA-8	2.7**	40.4**	62.4	9.1**

Note: WT, wild-type; MA, mouse-adapted.

a Number of white blood cells (WBC) in whole blood, expresses as thousands of WBCs per microliter of whole blood.

b Mean percentage of leukocytes that are lymphocytes (LY), neutrophils (NE), or monocytes (MO) from 10 mice per group at 4 d.p.i.

*Statistical significance: * *P*<0.05 and ** *P*<0.01 compared to the values of wild type virus.

### Infection with the mouse-adapted viruses modulates cytokine and chemokine responses in mice

**Figure 7:**
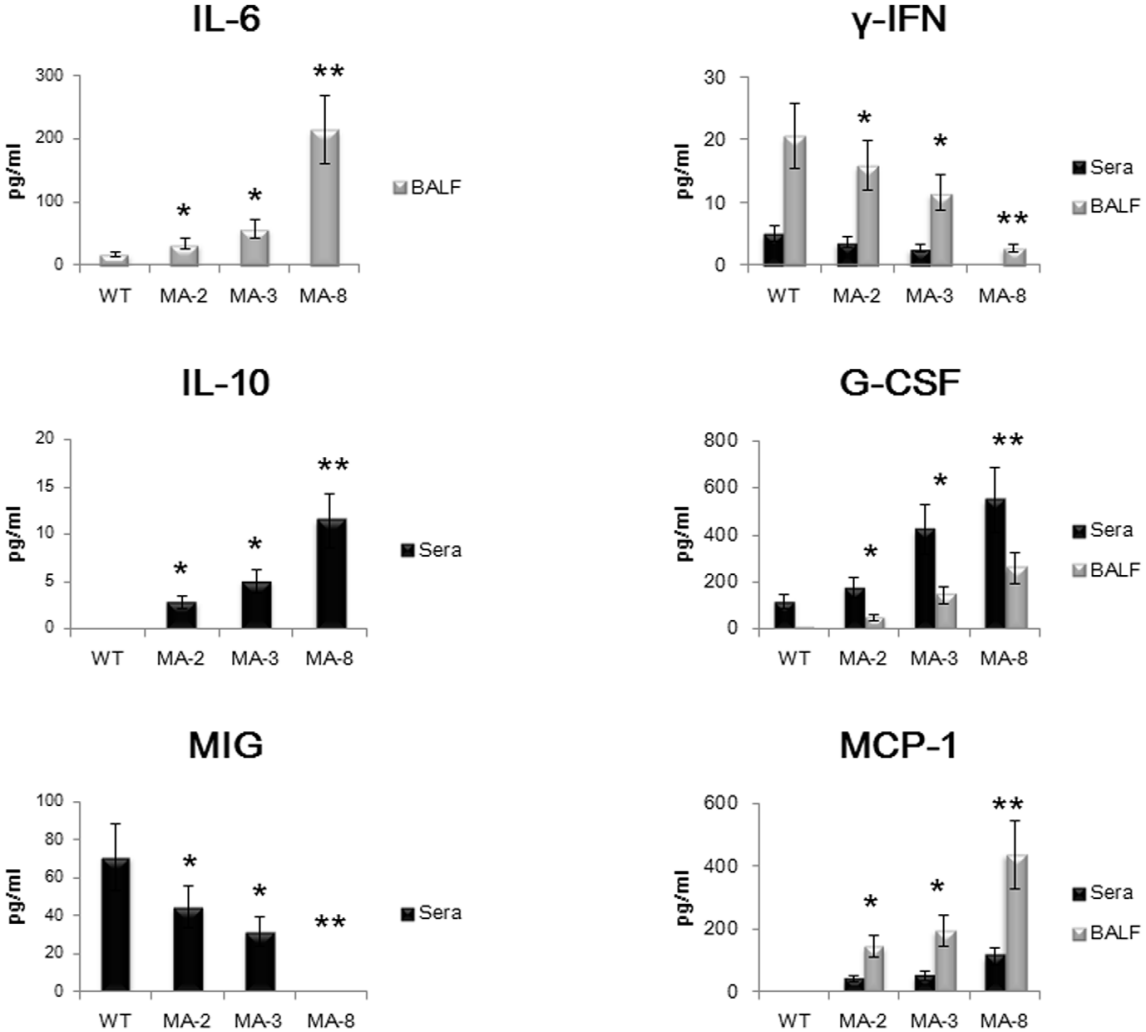
The cytokine and chemokine responses. The concentrations of cytokines and chemokines in BALF and sera of individual mice were measured quantitatively using Cytometric Beads Array. Data are expressed as mean ± SD of the concentrations of individual cytokines or chemokines in BALF or sera of each group of mice (n = 10 per group) from three separate experiments. There was no significant difference in the concentrations of other cytokines and chemokines tested (data no shown). * *P*<0.05 and ** *P*<0.01 vs. the values in the mice infected with wild type virus.

To further investigate if there was a correlation between severe disease and inflammatory cytokine and chemokine production in virus-challenged mice, the concentrations of cytokines and chemokines in sera and BALF were tested at 4 d.p.i using Cytometric Beads Array (Fig. 7). In comparison with that in the wild-type virus infected mice, infection with the mouse-adapted viruses, particularly with MA-8 (p<0.01), promoted significantly higher levels of IL-6, MCP-1, and G-CSF, but dramatically reduced levels of IFN-γ in BALF (*P*<0.05). Furthermore, infection with the mouse-adapted viruses, especially with MA-8 (p<0.01), induced higher levels of IL-10, G-CSF and moderate levels of MCP-1, but remarkably reduced levels of MIG in sera (*P*<0.05). However, there was no detectable IL-4, IL-12/IL-23p40, IL-13, IL-17, IL-21, GM-CSF, and KC in sera and BALF, and there was no significant difference in the concentrations of IL-6 in sera, IL-10 and MIG in BALF, IL-1α, IL-1β, TNF, MIP-1α, MIP-1β in both sera and BALF among the different groups of mice (data not shown).

## Discussion

Adaption is thought to be the driving force in evolution, during which organisms are selected in nature because of increased fitness conferred by beneficial mutations. Although mice are not naturally infected with seasonal H1N1 influenza viruses, many strains of mice can be infected with human strains of influenza viruses, which can be adapted to the mouse by serial lung passage [6]. In our study, a virulent strain of mouse adapted virus with virulence was produced following eight serial lung-to-lung passages in mice. Mice infected with the mouse-adapted viruses exhibited more extensive body weight loss and higher virus RNA loads in mouse BALF, higher virus titers, and more severe pathological changes in mouse lung tissues, compared with that in the mice infected with the wild-type virus.

Analysis of the potential molecular mechanism underlying the high virulence of the adapted viruses revealed that there were three mutations incrementally acquired at passage 2 (T89I), passage 3 (N125T), and passage 6 (D221G). The T89I mutation exists in another mouse lung adapted virus from A/USSR/90/77(H1N1), but not in natural strains of the human and animal influenza viruses [5], [10]. It is proposed that the Thr at residue 89 of HA1 is a potential glycosylation site, and the loss of glycosylation sites in HA protein represents a common theme during mouse lung adaptation [5]. The alteration has the potentials to impact on the specificity and affinity of receptor binding and the pH of HA mediated membrane fusion, contributing to the high virulence of adapted viruses [5]. However, it is still uncertain whether the increased virulence is caused by the T89I substitution only or the combinations of the three mutations. We are interested in further investigating the role of each mutation on the A/Brisbane/59/2007 backbone in the virulence of adapted viruses.

The HA of influenza virus binds to receptors possessing terminal *N*-acetyl neuraminic acid, also termed sialic acid (SA), linked to galactose through either α2-3 or α2-6 bonds (α2,3 SA or α2,6 SA, respectively). Subsequently, HA can internalize within endosomes, and mediate fusion and uncoating due to low-pH-induced conformational changes [34]. The HA of different types of influenza viruses preferentially binds to the linkages that are abundant in their natural host; for example, avian strains of viruses preferentially bind to α2,3 SA, but human strains to α2,6 SA [35]. The α2,3 and α2,6 SA receptors were expressed on the trachea, lung, cerebellum, spleen, liver and kidney, while the α2,6 SA receptors were expressed by epithelial cells of the cecum, rectum and blood vessels in the heart of BALB/c mice [36], [37]. However, we found that both wild-type and mouse-adapted viruses did not bind to the synthetic α2,3 SA receptors and there was no significant difference in the affinities of these viruses binding to the synthetic α2,6 SA receptors in *in vitro* receptor binding assay. These findings suggested that the mutations in the HA protein of the mouse-adapted viruses might not be critical for the binding of virus to the receptors. Furthermore, some studies have shown that mouse-adapted variants of human H1N1 and H3N2 viruses have altered pH of fusion [11], [31]. However, our *in vitro* hemolysis assay showed that mutations also did not change the host membrane fusion pattern of viruses. Although most of the mouse-adapted variants have elevated pH of fusion, the role of this property in virulence is not clear. Interestingly, the MA-8 displayed high virulence, but did not alter the pH of fusion, indicating that an increased pH of fusion was not necessary for the high virulence of mouse-adapted viruses. We are interested in further investigating how the mutations could contribute to the high virulence of mouse adapted viruses.

Characterization of virus replication indicated that there was no significant difference in the viral replication in MDCK cells between wild-type and mouse-adapted viruses. This observation was not surprising because a previous study has shown that the mutations in the matrix protein are responsible for increased replication of the mouse adapted A/FM/1/47 (FM) *in vitro* [13] and that the neuraminidase protein (NA) is responsible for the replication of A/WSN/33(H0N1) in MDBK cells [38]. Indeed, the mutations that affect viral virulence can be classified according to host-dependent and independent abilities to increase growth as well as changes in pathological properties [14]. Given that all the mutations were located in the HA of the mouse-adapted virus, these mutations might have little effect on modulating *in vitro* replication of the mouse adapted virus in MDCK cells.

The mouse-adapted virus showed significantly increased virulence and pathogenicity in mice. However, the receptor binding, the pH of HA mediated membrane fusion, and the *in vitro* replication in MDCK cells of mouse-adapted viruses were similar to that of the wild-type virus. These data suggest that the increased virulence and pathogenicity of mouse-adapted viruses may be attributed to their high immunogenicity that induces strong inflammation in the mice. Evidentially, infection with the mouse-adapted viruses, particularly with MA-8, induced severe lymphopenia in mice. These findings were similar to that in previous observations in humans infected with avian H5N1 or with triple-reassortant swine influenza [32], [33]. Similarly, mice infected with H5N1 and reconstructed 1918 H1N1 viruses developed severe lymphopenia [39], [40], which is likely associated with redistribution of lymphocytes that migrate into the inflammatory sites. Furthermore, hypercytokinemia can induce hemophagocytic syndrome that can result in pancytopenia due to excessive pan-phagocytosis [41], [42]. However, the precise mechanisms underlying the lymphopenia remain to be further investigated.

Secondly, we found that infection with the mouse-adapted viruses, particularly with MA-8, promoted high levels of pro-inflammatory cytokine and chemokine production in mice. Indeed, high levels of pro-inflammatory cytokines in nasal fluids and plasma are associated with severities of diseases in human volunteers infected with influenza virus, and strong cytokine responses, such as “cytokine storm” are commonly observed in cases infected with highly pathogenic influenza viruses, including recent H5N1 fatalities [43], [44], [45]. The high levels of pro-inflammatory cytokines in the mice further indicated high virulence and pathogenicity of the mouse-adapted virus. Interestingly, we detected significantly higher levels of IL-6, G-CSF, and MIP-1 in BALF and IL-10 in sera, but lower levels of IFN-γ and MIG in BALF and sera of the mice infected with mouse-adapted virus, respectively.

High levels of IL-6 have been detected in ferrets infected with high virulent H1N1 [46] and in some cases with severe clinical symptoms [43], [47]. IL-10 is a negative regulator of innate and adaptive immunity, and high levels of serum IL-10 may act as a feedback regulator of virus infection-induced inflammation [48]. High levels of MCP-1 in BALF may be responsible for recruiting inflammatory infiltrates in the lungs and have been detected in cases with H5N1 and 2009 pandemic H1N1 virus infection and in sera of macaques infected with 1918 H1N1 virus [49], [50], [51]. High level of G-CSF was found to be significantly overexpressed in sera and BALF from mice in which H3N2 influenza virus infection is followed by superinfection with S. pneumonia serotype 3, suggesting that G-CSF is a major contributor to synergistic exacerbation of disease leading to fatal infection [52]. However, our data presented here are the first report about the upregulation of G-CSF in mice with severe disease following simple infection with virulent influenza virus. Furthermore, studies based on ferrets showed that influenza virus strains causing severe disease were characterized by a lesser induction of type I and II interferons [46], and monokines induced by IFN-γ (MIG) have been shown to exhibit direct antiviral properties [53] and to be essential in the development of a protective Th1 response against viral infection in the central nervous system [54]. Thus, the down-regulations of IFN-γ and MIG may be critical factors for inducing severe pathogenicity in host animals or patients by some virulent influenza viruses. These data suggest that altered levels of IL-6, IL-10, G-CSF, MCP-1, IFN-γ, and MIG may be indicators of the progression to more severe disease caused by influenza viruses.

Apparently, mouse-adapted viruses clearly elicit a much greater immune response than the wild-type virus, which may be mainly caused by the mutations in the HA protein, and change the ability to replicate in mouse lungs. However, whether the mutations have increased the intrinsic immunogenicity of the virus is still under investigation. Characterization of immune response of mice against virus like particles (VLP) or recombinant protein would be valuable to determine the immunogenicity of wild-type and mutated HA proteins.

In conclusion, we obtained a lethal mouse-adapted seasonal H1N1 virus following serial lung-to-lung passages of wild-type virus in mice, and found that the mouse-adapted viruses had mutations in the HA protein. Furthermore, the mouse-adapted viruses had properties of receptor binding, the pH of HA mediated membrane fusion, and the *in vitro* replication in MDCK cells, similar to that of wild-type viruses, but it also had high virulence and pathogenicity in mice. Evidentially, infection with the mouse-adapted virus induced severe lymphopenia and strong pro-inflammatory cytokine responses in mice. Apparently, mouse adaption of the virus may change the ability of virus to replicate in mouse lungs, which induces strong immune responses and inflammation in mice. Therefore, our findings may provide new insights into understanding the mechanisms of mouse adaption and cross-species transmission of the human H1N1 viruses as well as the pathogenesis of highly virulent influenza in humans.
